# Standardize sub-speciality training to survive as a super-speciality

**DOI:** 10.4103/0970-0358.73422

**Published:** 2010

**Authors:** Surajit Bhattacharya

**Affiliations:** Editor, IJPS, Sr. Consultant Plastic Surgery, Sahara Hospital, Lucknow, India. E-mail: surajitb@sancharnet.in

India, the land of *Sushrutha*, has long been a place of dynamic development in plastic surgery, adapting to differing cultures, traditions and medical and surgical philosophies. Recently, these changes have been even more striking and rapid. Microsurgery, tissue expansion, craniofacial surgery, aesthetic surgery and the spin-offs of these techniques have made permanent changes in the care of trauma, treatment of malignancy, management of congenital anomalies and in aesthetic surgery.

Like all other fields of medicine, *plastic surgery* too is in a constant flux and state-of-the-art care is continuously being revisited. There is an urgent need for certificate of accreditation of the various sub-specialities, namely burns, hand surgery, cranio-maxillofacial surgery, microsurgery, reconstructive oncology and aesthetic surgery. The idea is not to monopolize, but to set standards and to give a structure to the training programmes. The process of training/certification/accreditation needs to be handled, not on whims and arrogance of personalities, but with a studied perseverance and a holistic approach, by patiently weaving joint efforts along with allied specialities. How can we think of running a certification programme in cranio-maxillofacial surgery without a neurosurgeon or an orthodontist? How can we dream of accreditation in trauma and microsurgery without an orthopaedic input?

While efforts should continue to improve training facilities in medical colleges, the vibrant private sector should take up the responsibility of training of the teachers in the government medical colleges in these various technology-based sub-specialities. Financial constrains, which the government institutions invariably face, prevent them from acquiring the state-of-the-art technology whether it is endo-surgical equipments or lasers. Teachers not exposed to these latest technologies are imparting a training which is far from recent. It is the primary responsibility of tertiary care centres to train teachers. One has to bear in mind that there is no contradiction between delivering plastic surgery to the masses and training the trainees and the trainers in sub-specialities that thrive on the state-of-the-art technology. In India we should refrain from ‘knee-jerk’ reaction of sacrificing either of them. A balance has to be struck to achieve the best of both the worlds so that one day we can use these now very expensive technologies to treat our not so affluent patients.

We must recognize that change is the only constant; a shift in paradigm is inevitable, and with time, science and technology will continue to evolve in an inherent attempt to balance our requirements. Strengthening the various sub-specialities will only strengthen our super-speciality and popularize it. Remember we are blessed with a name ‘Plastic’ that neither signifies an age group nor a gender or an organ or a disease and so not only the ignorant public but even some of our own colleagues consider us to be only cosmetic surgeons. The fact that there is aesthetics in every reconstruction we perform is almost as alien to them as the music of Mozart, Bach, Beethoven, Chopin, Brahms, Handel, Vivaldi, Strauss, Grieg and Tchaikovsky. Strong and vibrant sub-specialities will help them to appreciate the tenor and develop a taste for the symphony that is Plastic Surgery today!

In the present scenario, the number of training institutions for *plastic surgery* and trained plastic surgeons are already far below our national requirement and the addition of the units offering DNB are most welcomed. But this expansion of the mother speciality should not halt the development of the sub-specialities and the two can continue in the same hospital and in hospitals of different cadre side by side.

The fellows registered for training in these sub-specialities should have a recognized M.Ch./DNB degree. If the infrastructure is still not in place they can spend some time in centres of excellence elsewhere in the country. Multi-centric training has proven benefits and it invariably widens the horizon of the trainees. This approach will ensure that the sub-specialist cadres are developed within the existing infrastructure, till the time the units themselves become self-sufficient. Trainees should be trained both in public and private sector hospitals, so that they are not only exposed to the latest gadgets but they also come across a wider cross section of the population and are more market-ready.

Examinations too need to be revisited. They should be structured in such a way that instead of one final examination there are periodic appraisals of the trainee’s fund of knowledge, communication skills and intellectual integrity. Due importance should be given to research and publication and a trainee should not be allowed to appear in the final examination if his/her research is not published.

A candidate upon successfully qualifying the sub-speciality training should be able to:

- Offer to the community, the current quality of ‘standard of care’ in hand surgery, burn surgery, trauma surgery, microsurgery, cranio-maxillofacial surgery, reconstructive oncology or aesthetic surgery.
- Periodically self-assess his or her performance and keep abreast with ongoing advances in the field and apply the same in his/her practice.
- Be aware of his or her own limitations to the application of the sub-speciality in situations which warrant referral to more qualified centres or individuals.
- Apply research and epidemiological methods during his/her practice. The candidate should be able to present or publish work done by him/her.
- Contribute as an individual/groups towards the fulfilment of national objectives in various fields like leprosy, burns and cleft lip and palate.
- Effectively communicate with patients or relatives so as to educate them sufficiently and give them the full benefit of informed consent to treatment and ensure compliance.

All training programmes across the country need to be standardized. Sound academic is the backbone of quality output. The cutting-edge research in training programmes is integral to achieving these goals. The sheer numbers of our patients provide us a challenge and an opportunity to innovate, refine and simplify the management of complex reconstructive and aesthetic problems. This can be achieved by employing state-of-the-art technology, both in research and interventions, and enable us to lead the campaign at global pedestals.

The Association of Plastic Surgeons of India needs to spend quality time of its best brains in formalizing the organization and the curriculum of the training process. The ultimate goal of this august effort should be to continue to assure that hand surgeons, burn surgeons, cranio-maxillofacial surgeons, trauma surgeons, microsurgeons and aesthetic surgeons are of the highest attainable quality that will optimize the surgical health of Indians. Never before have so many simultaneous internal and external forces appeared on the horizon that have the collective potential of influencing the quality of future plastic surgeons-a declining applicant pool, the generation-Y factor of mass exodus towards lucrative cosmetic surgery, medical economics, lengthy training for specialization, poor initial returns, reliance on co-specialities, poaching by non-/semi-qualified cosmetologists and a non-standardized training and evaluation programme. The time is right to strike as the iron is red hot.

